# Assessment of Different Distraction Behavioral Methods in Pediatric Dental Clinic: A Systematic Review

**DOI:** 10.7759/cureus.42366

**Published:** 2023-07-24

**Authors:** Mohammad Dahlan, Rafal Alsaywed, Raghad Alamoudi, Amal A Batarfi, Omama Y Basodan, Yara Gazzaz, Yasmeen A Alqarni, Ahmed Alamoudi

**Affiliations:** 1 Pediatric Dentistry, North Jeddah Speciality Dental Center, Jeddah, SAU; 2 General Dentistry, King Abdulaziz University, Jeddah, SAU; 3 Oral Biology, King Abdulaziz University, Jeddah, SAU

**Keywords:** pediatric patients, pedodontics, dental fear and anxiety, pediatric dental clinic, children behaviors, distraction methods

## Abstract

Dental anxiety is one of the main problems dentists may face during the treatment of pediatric dental patients; therefore, clinicians tend to perform different behavior management techniques to reduce dental anxiety in children. This review aimed to systematically compare and evaluate the published literature regarding the effects of distraction techniques on anxiety, pain perception, and patient experience during dental practice. A detailed electronic search was conducted on 3 databases including PubMed, Google Scholar, and Cochrane Library. The databases were searched for articles published in the English language between 2015 and 2022. Among 102 studies, 27 studies fulfilled the criteria of eligibility and were included in this study to be analyzed. Numerous approaches have been proposed for the reduction of dental anxiety, out of which the use of audio-visual aids and instruments, active distraction such as tablets, smartphones, and virtual reality glasses showed governance in decreasing the children’s anxiety followed by cognitive and behavioral methods.

## Introduction and background

Commonly dental anxiety and dental fear occur in children [1]. It is defined as an emotional reaction to constant distress associated with dental treatment and procedures [2,3]. It can involve the emotional, behavioral, physiological, and cognitive components that may vary between personalities [2,3]. The primary function of fear and anxiety is associated with the activation of the sympathetic system and the patient's reaction. The patient perceives this as a signal of threat and danger due to unusual surroundings, noise production in dental setups, potentially painful or invasive procedures, and previous negative experiences [4-7]. Pain has also a psychosomatic factor centered on the extent of responsiveness focused on the noxious stimulus modifying the discomfort and pain [8]. The incidence and prevalence of dental anxiety and fear range from 6% to 42% among children of different ethnicities and backgrounds [8].

The management of uncooperative children with a fear of pain and dental anxiety can be a demanding job for the health care professionals as well as for the parents/guardians and also for the children [9]. Many psychosomatic interventions have been utilized for the management of distress, anxiety, and dental pain that aids in the development of coping mechanism [9]. The dental care providers used different techniques and therapies to manage patient’s distress and anxiety such as traditional behavior management, music, virtual reality, magic tricks, eyeglass systems, in-ear headphones, gaming consoles, videos, hypnosis, and systematic desensitization [9-11].

In current years, many studies have been published for the evaluation of the efficiency and beneficial effects of distraction methods and techniques, which can be employed for the behavioral management of patients and their parents/guardians [8]. A systematic review conducted by Prado et al. only included 20 studies and found low certainty of evidence that distraction techniques reduced dental anxiety [8]. Another study by Allani et al. only focused on the effectiveness of distraction techniques rather than the usefulness and long-term benefits of the techniques [11]. Knowledge of the usefulness of distraction techniques may be useful for the dental team to manage anxiety and create a pleasant environment to improve the children’s dental experience [1]. To date, no researcher has critically reviewed up-to-date literature on all methods of distraction techniques during dental treatment. Thus, this study was aimed at the assessment of distraction techniques in the reduction of children's pain perception and anxiety in dental setups.

## Review

Research methodology

Reporting Guidelines

A systematic review in accordance with the preferred reporting items for systematic review and meta-analyses (PRISMA) was reported.

Eligibility Criteria

The inclusion criteria of the study were published in the English language and included original research, randomized control trial, and comparative studies based on distraction methods used to manage dental anxiety during the dental treatments; patients not older than 12 years of age with normal hearing and vision; not undergoing any emergency treatment. Letter to the editorials, incomplete trials (whose results were not published yet), case reports, pilot studies, books, gray literature, studies based on any other type of behavior management, and dental patients who were medically compromised (physically or mentally disabled) were excluded from this research.

Database Search

An electronic search from three databases; PubMed, Google Scholar, and Cochrane Library was performed between 2015 and 2022. A manual search of the references list of included articles was also conducted to identify any other studies that might have been missed in the electronic search. Finally, all references were exported into a pre-defined Excel sheet in a comma-delimited format for manual screening.

Search Strategy

According to the PRISMA guideline, this review was conducted through electronic database searching using PubMed, Google Scholar, and Cochrane Library. The Boolean operator “AND” was used to merge the search themes for the database search. The terms “Behavior” AND “Anxiety” AND “Distraction” AND “Pediatric Dentistry AND “Children” were utilized to quantify the distraction methods used to treat dental pain and anxiety in children. The literature was searched for papers published in the English language, between 2015 and 2022. The summary of the search strategy is shown in Table 1.

**Table 1:** Search strategy and number of articles obtained.

Electronic Databases	Search Strategy	Number of Articles
PubMed	((Behavior OR Anxiety OR dental anxiety OR phobia OR dental fear AND (distraction) AND (Pediatric Dentistry OR dental care OR dentistry OR dental treatment) AND (Children))	82
Google Scholar	((Behavior OR Anxiety OR dental anxiety OR phobia OR dental fear AND (distraction) AND (Pediatric Dentistry OR dental care OR dentistry OR dental treatment) AND (Children))	67
Cochrane library	((Behavior OR Anxiety OR dental anxiety OR phobia OR dental fear AND (distraction) AND (Pediatric Dentistry OR dental care OR dentistry OR dental treatment) AND (Children))	16

Study Selection

Two independent reviewers (MD and RA) screened the articles based on the inclusion criteria. The screening is in phases. The first phase of the screening is going through the abstracts and titles of the studies in order to select relevant articles. Studies that did not meet the eligibility criteria were excluded. The second phase of the screening is the evaluation of the full text of the remaining articles based on the same inclusion criteria. Articles that did not meet the exclusion criteria were excluded during the second phase. The remaining articles were retained for quality assessment. Disagreement between the researchers was settled by means of discussion with the other authors until a consensus was reached.

Data Extraction

Data were extracted from the included studies into a pre-defined Excel sheet. The two reviewers (MD and RA) extracted the data twice to minimize errors that may occur, in case of any discrepancies, consensus was reached. The following data were extracted from the included studies; author's first name, year of publication, study type, journal name, participants, age in year, distraction method or techniques reported, study results, and conclusion.

Quality Assessment of the Included Studies

The quality of the included studies was assessed by the reviewers using the Cochrane Collaboration’s Risk of Bias tool (Version 5.4) comprises seven domains: sequence generation, allocation concealment, blinding of participants and personnel, blinding of the outcome, incomplete outcome data, selective outcome reporting, and other bias. Each of the domains is made up of “high”, “low” and “unclear” risks of bias. The quality assessment of the non-randomized study was assessed through the Newcastle Ottawa tool.

Synthesis of Results

The data extracted from the included studies were analyzed in a descriptive approach. Data were presented in mean and standard deviation. We could not perform a meta-analysis due to methodological heterogeneity.

Results 

Study Selection

**Figure 1:**
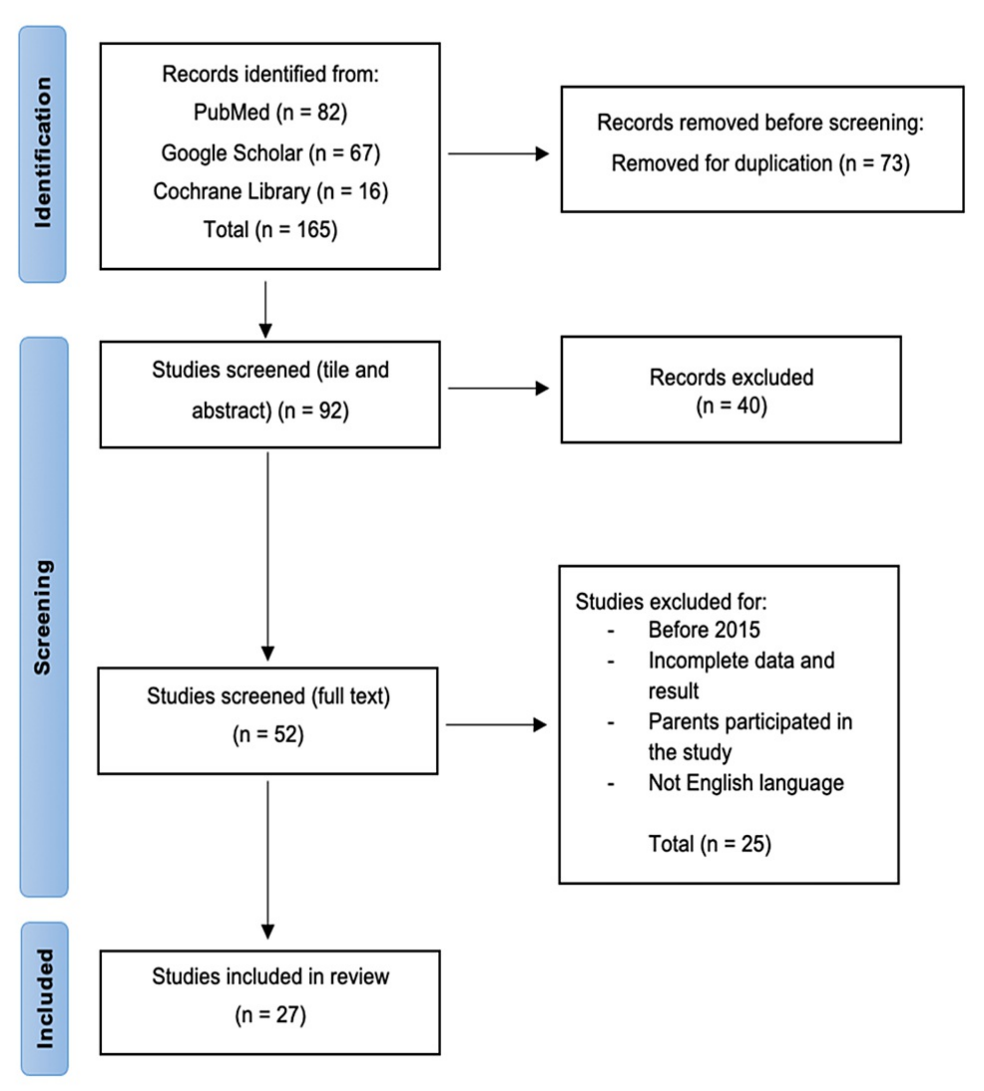
PRISMA flow diagram of the literature search results.

The three databases generated 165 articles, 82 from PubMed, 67 from Google Scholar, and 16 from Cochrane Library. The two independent reviewers (MD and RA) performed screening and 27 relevant articles met the eligibility criteria and were retained for the systematic review. PRISMA flow diagram shows the selection process as presented in Figure 1.

Quality Assessment of Individual Studies

**Figure 2:**
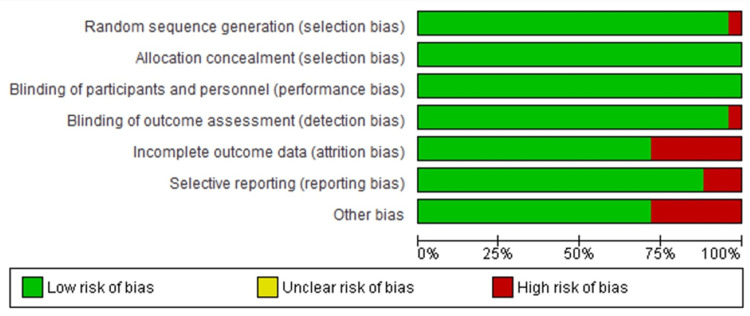
Summary of quality assessment from risk of bias tool.

The results of the risk of bias assessment using Cochrane Collaboration’s Risk of Bias tool were presented in Figure 2. The main problem was identified in the incomplete outcome of data in these studies; Aditya et al., Al-Halabi et al., Chaturvedi et al., Rajeswari et al., Kaur et al., and Hegde et al. [12-17]. Also, selective reporting was another identified issue in some of the studies [14,18,19]. Another risk of bias identified is sample size selection, most of the studies failed to randomize the participants into the group and also failed to present a power test for the sample size calculation [20-22]. According to Newcastle Ottawa, the prospective study's [23] shortcoming was a lack of ascertainment of exposure and inadequate follow-up. Whereas for the cross-sectional study [24], a high risk of bias was identified for the outcome assessment and adequate follow-up (Table 2).

**Table 2:** Measurement of risk of bias of (non-randomized-cross-sectional study and prospective study) using the Newcastle-Ottawa scale.

Studies	Selection				Comparability	Outcome		Score
	Representativeness of the expose cohort	Selection of non-expose cohort	Ascertainment of exposure	Precision of exposure	Confounding controlled	Outcome assessment	Adequate follow up	Total
Sharma et al., 2021 [24]	*	*	*	*	*	--	-	5
Debs et. al., 2017 [23]	*	*	-	*	*	*	-	5

Study Characteristics

Twenty-seven studies related to pediatric dentistry published between 2015 and 2022 were included in the review. Out of which 25 were RCTs, one prospective study, and one cross-sectional study [23,24]. The sample size ranged from 28 in the study of Ghadimi et al. [24] to 123 in Shekhar et al.'s study [25]. The age of participants ranged from three years old to 12 years old [26,27]. Nine distraction techniques were identified: audiovisual, aroma therapy, virtual reality, video eyeglasses, intellectual color game, vibration, bubble blower, magic trick, and fidget spinner, kaleidoscope [13-18,20,21,24,25,27-36]. All the distraction techniques used were presented in Table 3. The full details characteristics of included studies can be found in Table 4.

**Table 3:** Results of different distraction methods reported in the included studies.

Identified Distraction Method	References	Number
Audiovisual Vs Others (Muted Video)	Jafarimo et al., 2022 [18], Navit et al., 2015 [27], Kaur et al., 2015 [16], Guinot et al., 2021 [19], Shetty et al., 2019 [21], Felemban et al., 2021 [28], Halabi et al., 2018 [13], Khandelwal et al., 2018 [29], Chaturvedi et al., 2016 [14], Delgado A et al., 2021 [10], Rao et al., 2019 [30], Shekhar et al., 2022 [25], Sharma et al., 2021 [24], Al-Khotani et al., 2016 [31], Pande et al., 2020 [32], Rajeswari et al., 2019 [15], Mahajan et al., 2022 [33].	17
Music	James et. al., 2021 [34], Gupta et al., 2017 [26]	2
Virtual Reality	Greeshma et. al., 2021 [35], Ghadimi et al., 2018 [20]	2
Magic Trick	Thosar et. al., 2022 [36]	1
Video Eyeglasses	Garrocho-Rangel et al., 2018 [20]	1
Intellectual Colored Games	Debs et. al., 2017 [23]	1
Vibration	Hegde et. al., 2019 [17]	1
Fidget Spinner, Kaleidoscope	Aditya et al., 2021 [12]	1
Bubble Blower	Bahrololoomi et al., 2022 [37]	1

**Table 4:** Characteristics of included studies.

	Authors/Year of Study	Journal Name	Study Design	Participants Age (Year)	Sample Size	Distraction Method/ Technique	Study Results	Conclusion
1.	Jafarimofrad et al./2022 [18]	Dental Research Journal	RCT	4-7	60	Audiovisual and muted video	The study measured the pulse rate (PR), face pain rating (FPR) and sound eye motor pain (SEM) scale during the dental procedures. The audiovisual distraction method result showed statistically significant (P < 0.05) result in comparison with muted video distraction method	Audiovisual distraction (AVD) method was beneficial in reducing the anxiety and pain of children while mute-video distraction (MVD) method was not effective
2.	Navit et al./2015 [27]	Journal of Clinical and Diagnostic Research	RCT	6-12	150	Audio only (instrumental music, the audio stories, movie songs and the musical nursery rhymes	Anxiety was assessed using the Venham's picture test (VPT), Venham's clinical rating scale (VCRS) and pulse rate. The significant difference (p<0.001) was observed in the pulse rate while the VPT and VCRS differences were not statistically significant(p>0.05)	Audio stories were found to be more effective in anxiety reduction in children
3.	James et al./2021 [34]	International Journal of Clinical Pediatric Dentistry	RCT	6-8	150	Music and aroma therapy	In comparison of music and aroma therapy with control group, significant reduction in anxiety levels, respiratory and pulse rate was detected with reference to Facial image scale (FIS) and VPT	Both the techniques produced the better results. However, the music distraction produced was found to be more effective.
4.	Greeshma et al./2021 [35]	International Journal of Clinical Pediatric Dentistry	RCT	6-8	90	Virtual reality distraction, audio distraction and tell-show-do	FIS showed statistical reduction in anxiety (p <0.01), using all these distraction techniques	Virtual reality (VR) was found to be most effective distraction tool in comparison with audio and tell show do methods
5.	Gupta et al./2017 [26]	The Journal of Contemporary Dental Practice	RCT	3-7	60	Upbeat music and relaxing music	Pain, heart rate and behavioral change were measured in the study. The results showed music did not produce any significant results	Music distraction was found to be not effective in this study
6.	Kaur et al./2015 [16]	Journal of Indian Society of Pedodontics and Preventive Dentistry	RCT	4-8	60	Audio and audiovisual	Dental fear schedule sub scale-short form (DFSS-SF) and heart rate were measured to assess the anxious children behavior. Significant difference at level of 0.05% were detected in audiovisual group and visual group from the control group	Audiovisual mode of distraction technique was found to be a most beneficial to manage the anxiety of children
7.	Guinot et al./2021 [19]	European Journal of Paediatric Dentistry	RCT	6-8	84	Active audiovisual (play station video games) and passive audiovisual (cartoon film)	Significant differences were reported for pain between the experimental (P=0.013) and the control (P=0.016) groups	Both active and passive distraction methods were adapted by the patients
8.	Shetty et al./2019 [21]	The Journal of Clinical Pediatric Dentistry	RCT	5-8	120	Audiovisual (virtual reality)	Anxiety and pain in children was significantly decrease (p=0.002, p<0.001) respectively by using virtual reality methods	This can be practiced as an effective behavior adaptation technique in children during invasive procedures of dental treatments
9.	Felemban et al./2021 [28]	BMC Oral Health	RCT	6-12	50	Audiovisual (virtual reality)	Regardless of the distraction, the females and the young age patients reported high mean face, legs, activity, cry and consolability (FLACC) scores (P=0.034 and P=0.004). Patients with high pulse rate at baseline, reported high mean Wong– Baker Faces (WBF) score (P=0.031 and P=0.010)	Female subjects and the younger age and female dental patients were found to be more likely to appear with higher pain scores regardless of the distraction tool used in treatment
10.	Al-Halabi et al./2018 [13]	Anaestha, pain & intensive care	RCT	6-10	102	Audiovisual (virtual reality box and tablet device with wireless headphone)	Non statistical significant (p = 0.536, p = 0.454 ) were measured for anxiety via the Wong–Baker Faces and FLACC scale respectively. But the anxiety and pain were statistically significant reduced (p = 0.043) when measured the pulse rate	VR box was found to be not effective while tablet device was helpful
11.	Khandelwal et al./2018 [29]	The Journal of Contemporary Dental Practice	RCT	5-8	400	Audiovisual (cartoons and animated clips) and tell show do methods	The statistical reduction in anxiety (p < 0.05) was measured by using distraction techniques	Combination of both the techniques produced more beneficiary effect in relieving the anxiety. AVD showed more better results than tell-show-do (TSD)
12.	Chaturvedi et al./2016 [14]	Journal of International Oral Health	RCT	6-10	40	Audiovisual (with and without wearing eyeglasses)	Pain and anxiety reduction were recorded via the Wong-Baker pain rating scale (WBPRS) and visual analogue scale (VAS) scoring. Statistically significant reduction (P < 0.05) in anxiety was measured for group with audiovisual eye glasses. The pulse rate measured by pulse oximeter was also highly statistically significant in audiovisual eye glasses group	This technique was proved to be a good option in child’s anxiety management
13.	Delgado et al./2021 [10]	Clinical and Experimental Dental Research	RCT	4-6	100	Audiovisual	The study revealed a high statistically significant (p < 0.0001) result on Frankl scale and definitely positive behavior (91.8%) evaluated from the pre and the post treatment.	Despite of the children’s subjective expression, AUD was helpful as a distraction tool for behavior management
14.	Rao et al./2019 [30]	International Journal of Clinical Pediatric Dentistry	RCT	6-10	30	Audiovisual (virtual reality)	The results showed statistically significant reduction (p < 0.0001) in the anxiety and pain perception of children due to the usage of virtual reality tool	Virtual reality tool helps in distraction of children’s anxiety level
15.	Shekhar et al./2022 [25]	European Archives of Paediatric Dentistry	RCT	8-12	123	Active (stress ball) and passive (audiovisual)	The parameters of the study were the dental anxiety, pain and behavior. Statistically significant reduction was measured in anxiety within the groups but not between the groups. And no statistically significant differences were observed for pain and behaviour scores	Dental anxiety was improved by distraction techniques
16.	Ghadimi et al./2018 [22]	European Archives of Paediatric Dentistry	RCT	4-5	28	Visual (cartoon and tell show do)	In comparison with the TSD, cartoons significantly reduced (p-value<0.001) the anxiety but FBRS were not statistically significant (p-value=0.24)	Heart rate and the self-reported were reduced by the distractions
17.	Sharma et al./2021 [24]	International Journal of Clinical Pediatric Dentistry	Cross-sectional Study	4-8	97	Audiovisual (verbal method, video eyeglass/earphone system and digital screens)	The highest mean FLACC score for pain was recorded in verbal group (6.88), than digital screens (3.67) and least in video eyeglass/earphone (1.94)	Video eyeglasses/earphone was found to be more helpful in reduction of children’s anxiety
18.	Al-Khotani et al./2016 [31]	Acta Odontologica Scandinavica	RCT	7-9	56	Audiovisual	The results showed significant differences (p = 0.029) in experimental and the controlled group by using the modified Venham's clinical ratings of anxiety and cooperative (MVARS) scores. During the treatment MVARS scores were significantly decreased (p = 0.04) in experimental group and the pulse rate was significantly increased (p = 0.02) in controlled group	AVD methods showed positive responses in reduction of anxiety and fear in children
19.	Garrocho-Rangel et al./2018 [20]	European Journal of Paediatric Dentistry	RCT	7-9	56	Video eyeglasses/earphones system	No statistical differences were recorded with the use of eyeglasses/earphones system (VEES)	VEES distraction was not effective
20.	Pande et al./2020 [32]	Journal of Indian Society of Pedodontics and Preventive Dentistry	RCT	5-8	60	Audiovisual (virtual reality, tell show do and video game)	Facial image scale (FIS), blood pressure and pulse rate were assessed to manage the child’s anxiety. After the dental treatment, statistically significant differences (P = 0.000) were measured in all three groups while VR group sowed the most reduction in the anxiety of children	VR alone was very helpful and TSD alone was the least beneficial in managing anxious child
21.	Rajeswari et al./2019 [15]	International Journal of Clinical Pediatric Dentistry	RCT	6-10	45	Audiovisual, cognitive behavioral play therapy and tell show do	Statistically significant reduction in anxiety scores were detected in all the three groups (p = 0.001). However, high score in anxiety reduction was measured in cognitive behavioral play therapy (CBT) (p = 0.0)	CBT was found to be most effective method in children’s anxiety reduction
22.	Debs et al./2017 [23]	Journal of International Society of Preventive and Community Dentistry	Prospective Clinical Study	5-11	41	Intellectual colored games	Significant reduction (P < 0.001) was observed in gag reflex and anxiety of children associated with the dental procedure by using the FIS and gag reflex grading scores	Intellectual colored Games (ICG) distraction tool helped in the management of child’s behavior
23.	Hegde et al./2019 [17]	J Dent Anesth Pain Med	RCT	6-11	30	Vibration	The results represented the lower FLACC and pain rating scores with a decrease in the pulse rate by busing the device	It helped more in the management of the patients than the conventional method
24.	Mahajan et al./2022 [33]	International Journal of Clinical Pediatric Dentistry	RCT	5-9	60	Audiovisual (PlayStation gaming console) and print format entertainment (favorite comic)	Significant differences (p < 0.05) were measured in self-reported anxiety and pain by using the VPT and WBF scales in children of 5-9 years	Distraction tools were effective in the management of patient’s anxiety
25.	Aditya et al./2021 [12]	Heliyon	RCT	6-9	102	Fidget spinner, kaleidoscope and virtual reality	The mean VPT scores were highly significantly reduced (p < 0.001) in experimental groups but the oxygen saturation levels results were statistically non-significant (p > 0.05)	Fidget spinner, kaleidoscope, and virtual reality seem to be effective distraction methods and can be recommended as effective approaches to help alleviate children's dental anxiety during inferior alveolar nerve block procedures
26.	Bahrololoomi et al./2022 [37]	Hindawi, Pain Research and Management	RCT	7-10	35	Bubble blower	No significant differences were found in children for Facial image Scale (FIS). However, statistically significant (P value <0.05) results were obtained for the Wong-Baker faces pain rating (WBFPS) and FLACC scales by using a bubble blower for the breathing exercise.	The bubble blower used for the breathing exercises can be used as a beneficial and effective distraction method to reduce the moderate to severe anxiety and pain during the invasive dental procedures
27.	Thosar et al./2022 [36]	Cureus	RCT	4-11	30	Magic trick and audiovisual	A decrease in the level of anxiety was seen in both the groups (audiovisual along with magic tricks) by using Venham’s picture test. The pulse rate and the blood pressure (hemodynamic parameters) of the children were reduced throughout the 2^nd^ appointment, while the oxygen saturation level was seen to increase.	The study result showed that audiovisual methods and magic tricks were effective in reducing the anxiety in dental patients.

Synthesis of Results

A detailed explanation of the results according to different distraction behavior methods on pediatric patients at the dental clinic were presented in Table 3. The findings and conclusion from individual studies were presented in Table 4.

Audiovisual Distraction Method in Comparison to Other Distraction Method

In a study, children aged four years to seven years were randomized into the audiovisual group and muted video group [18]. During the dental procedures, the PR, FDR, and SEM scales were measured, and they found a significant result in the audiovisual distraction method compared to muted video method [18]. Another study measured DSS-SF, heart rate, and behavioral change in children with the pediatric disease to assess the anxious children's behavior, the results showed a significant difference at a 5% level in the audiovisual group and visual group compared to the control group [16]. Khandelwal et al. compared the audiovisual to tell-show-do (TSD) method on children aged five years to eight years, cartoons and animated clips were used in the audiovisual groups, the study reported a statistical reduction in anxiety (p < 0.05) in both groups [29].

Chaturvedi et al. had 40 pediatric patients aged between six years and 10 years and were randomized into the audiovisual group, the first group was asked to wear eyeglasses, and the other group was without eyeglasses [14]. Pain and anxiety were measured in both groups using VAS and WBPRS scales, statistically significant reduction (p < 0.05) in anxiety was measured for the group with audiovisual eyeglasses [14]. The pulse rate measured by pulse oximeter was also highly statistically significant in audiovisual eyeglasses group [14]. A similar study [24] used verbal method, video eyeglasses, and earphones in the first audiovisual group and digital screens in the second audiovisual group, the study recorded the highest mean FLACC score for pain in the verbal group (6.88), then digital screens (3.67) and least in video eyeglass/earphone (1.94).

Rajeswari et al. randomized 45 children into three groups; audiovisual, cognitive behavioral play therapy, and TSD group, statistically significant reduction in anxiety scores were detected in all three groups (p = 0.001) [15]. However, high score in anxiety reduction was measured in cognitive behavioral play therapy (p = 0.0) than in others [15].

Other Distraction Method in Comparison to No Distraction Method

A bubble blower was used for breathing exercises among 35 children aged seven years to 10 years [37]. The bubble blower can be used as a beneficial and effective distraction method to reduce the moderate to severe anxiety and pain during the invasive dental procedures (IANB), Bahrololoomi et al. recorded a significant result for the WBFPS and FLACC scales by using a bubble blower for the breathing exercise [37]. Magic tricks are another distraction technique that helped reduce the level of anxiety among children [36]. Thosar et al. compared magic tricks to an audiovisual using Venham’s picture test on children aged four years to 11 years old, the study found that the pulse rate and the blood pressure (hemodynamic parameters) of the children were reduced throughout the second appointment, while the oxygen saturation level was seen to increase in both groups [36]. Another technique is vibration which helped more in the management of the patients than the conventional method [17]. Moreover, intellectual Colored Games distraction tool helped in the management of child’s behavior [23].

Discussion

A major reason for the increase of dental caries and poor oral health in pediatric patients has reported to be dental anxiety [1]. Several methods have been proposed across the world to overcome this particular problem and ensure compliant visits to dentists [9-11]. Anxious pediatric patients can end up harming both themselves and the practitioner [5-7]. Moreover, the presence of anxiety can result in failure to complete the procedure in time, waste of materials and rescheduling of appointments [6].

The various methods that are used to curb dental anxiety includes the use of audio and visual aids, behavioral therapy and TSD methods during the treatment allowing the child to get treated in a calm and familiar environment [29,31,33]. Play stations or virtual reality devices have been more effective as compared to conventional methods of distraction such as voice modulations, jokes and changing topics of discussion [13]. The opposite of these facts has been reported in studies whereas school-based presentations and role-play have been found to be more useful as compared to TSD techniques [38].

A comparison between audio-visual and TSD technique showed that TSD is less productive than audio-visual aid [29]. This phenomenon has been supported by several studies which found audio-visual aid superior to other methods [38,39]. A significant improvement was noticed in the behavior of children aged between four and six years old who were exposed to audio-visual aid with a p-value of 0.001 as compared to the control group during dental procedures. However, no significant difference in pain expression was found [10]. The perception of pain in pediatric patients can also be significantly reduced as reported by several studies [33]. There has been considerable debate regarding whether audio-visual methods as a single modality are more effective or whether one of these techniques is preferred over the other [18]. It has been found that video distraction without sound does not prove to be useful in decreasing dental anxiety making audio a necessary component for distraction [18]. This is in contrast to the result reported in the study by Mishra et al. where no significant difference was found in the effectiveness of audio and audio-visual aids. Audio-visual aids if further divided into categories show audio aids comprising of stories to be more effective than rhymes or movie songs as reported in a study [15]. Dental anxiety can be considered a psychological phenomenon where the environment and the sounds of the dental clinic can disturb the pediatric patient resulting in an obvious reluctance to undergo dental procedures [40]. Activities that can distract the mind can be particularly useful in overcoming fear and anxiety [8,11]. The use of smartphones and virtual reality glasses transporting the patient to an imaginary world has been found to be instrumental in this regard [30]. Abbasi et al. in their study conducted on pediatric patients found smartphones to take a lead on dental songs and videos in distracting anxious patients [40]. A more constructive method of alleviating anxiety in pediatric patients includes the use of cognitive behavioral therapy which has superseded the effectiveness of audio-visual aid and conventional TSD technique [15]. In addition, several forms of advanced behavioral therapy have also been used [32]. This also includes the use of intellectual methods such as counting activities and identification games which have been found to be useful in overcoming anxiety in pediatric patients [23]. This finding has also been supported by several studies [41]. Recent advances in dentistry have led to the use of other innovative techniques such as music therapy and aromatherapy during dental procedures [34]. Studies have indicated that the use of music alone provides an insignificant improvement in dental anxiety [26]. However, the use of music and aromatherapy in combination has proven to be more powerful [42].

There are several dental procedures that can incite extreme anxiety in pediatric patients [2]. Innovative and efficacious methods need to be designed for such procedures. Studies have shown that breathing exercises using a bubble blower can be helpful in decreasing anxiety during administration of Inferior alveolar nerve block (IANB) [37]. Moreover, studies have shown audio-visual techniques to be effective and behavioral therapy has also been found to be profoundly helpful to curb anxiety during these procedures [43,44]. Menedez et al. found two techniques that are covering the patient’s eyes method and the hand-eye-mouth distraction strategy to be equally significant [45].

Limitations

The study encountered some limitations during the analysis and interpretation of data. The first limitation is the inability to conduct a meta-analysis due to the high percentage of heterogeneity among the included studies. Some of the included studies used the audio-visual distraction method as a control, while some studies used the audio-visual as an intervention [15,36]. A meta-analysis required studies with unrelated intervention and control groups. Another shortcoming was searching period limits, as the study addresses articles published between 2015 and 2023. Articles published before 2015 might be relevant to the present studies but were excluded from the review. Future researchers should try expanding the search limit to capture more articles as this may allow the conduct of meta-analysis.

## Conclusions

Dental anxiety is a major concern in the dental treatment of pediatric patients. Several methods to reduce dental anxiety have been proposed out of which the use of audio-visual aids, active distractions such as smartphones, and virtual reality show a clear dominance in reducing anxiety followed by cognitive and behavioral methods. There is considerable ambiguity in the method of judgment of both anxiety and its alleviation in patients, therefore requiring further research to be done in this area to develop a uniform method to evaluate anxiety levels and identify the most useful distraction techniques.
